# A Climatological Study of Scorpion Sting Incidence From 2007 to 2011 in the Dezful Area of Southwestern Iran, Using a Time Series Model

**DOI:** 10.1093/jisesa/ieu013

**Published:** 2014-01-01

**Authors:** Seyedeh Maryam Molaee, Kambiz Angali Ahmadi, Babak Vazirianzadeh, Seyed Abbas Moravvej

**Affiliations:** ^1^ Dezful University of Medical Sciences, Dezful, Iran; ^2^ Department of Biostatistics, School of Health, Ahvaz Jundishapur University of Medical Sciences, Ahvaz, Iran; ^4^ Department of Medical Entomology, School of Public Health and Infectious and Tropical Diseases Research Centre, Ahvaz Jundishapur University of Medical Sciences, Ahvaz, Iran; ^5^ Department of Plant Protection, College of Agriculture, Chamran University, Ahvaz, Iran

**Keywords:** scorpion sting, climatological parameter, time series model, auto regressive and moving average, Iran

## Abstract

Scorpion stings are a public health problem in south and southwest Iran. There is little information regarding climatological effects on incidence of scorpion stings in Iran. Therefore, the present systemic survey of scorpion sting data was conducted from the point of view of entomo-meteorological relationships and analyzed statistically for the Dezful area in Khuzestan, southwest of Iran. The time series analysis was implemented using MINITAB version 16 statistical software packages. In total, 3,755 scorpion sting files from the Dezful health centers were monitored from April 2007 to September 2011 in a time series analysis. The results showed that temperature had significant effects on scorpion sting. From the data of this study, it is concluded that the scorpion activity in Dezful County is a climatological-dependent phenomenon.

Scorpion stings are a public health problem in south and southwest Iran. Annually many people are exposed to scorpion stings, and some of them result in death or incurable problems. In Iran, 192,351 cases from 2001 to 2005 were reported in which 104 cases resulted in death ( Dehghani and Valaie 2005 ; Azhang and Moghisi 2006 ; Dehghani et al. 2010 , 2012 ). The highest mortality and number of the sting cases have occurred in Khuzestan, a southwest province of Iran ( Azhang and Moghisi 2006 ), and is an endemic health problem in Khuzestan ( Pipelzadeh et al. 2007 ).

Among the 1,500 scorpion species across the world, 25 species threaten human life ( de Roodt et al. 2003 ). The Iranian scorpion fauna consists of >51 species in 23 genera and 4 families, Buthidae, Scorpionidae, and Hemiscorpiidae. However, *Hemiscorpius lepturus* , which belongs to the Hemiscorpiidae family, is the most medically important scorpion in Iran ( Farzanpey 1987 , Lourenço 2001 , Lowe 2010 , Mirshamsi et al. 2011 ) ( Table 1 ).

**Table 1.** List of scorpion species occurring in Iran according to families of scorpions ( Mirshamsi et al. 2011 )

Buthidae
1. *Androctonus baluchicus* (Pocock, 1900)
2. *A. crassicauda* (Olivier, 1807)
3. *Apistobuthus susanae* (Lourenço, 1998)
4. *Buthacus macrocentrus* (Ehrenberg, 1828)
5. *Compsobuthus garyi* ( Lourenço and Vachon, 2001 )
6. *Compsobuthus jakesi* (Kovařík, 2003)
7. *Compsobuthus kafkai* (Kovařík, 2003)
8. *Compsobuthus kaftani* (Kovařík, 2003)
9. *Compsobuthus matthiesseni* (Birula, 1905)
10. *Compsobuthus persicus* (Navidpour et al. 2008)
11. *Compsobuthus petriolii* (Vignoli, 2005)
12. *Compsobuthus plutenkoi* (Kovařík, 2003)
13. *Compsobuthus rugosulus* (Pocock, 1900)
14. *Compsobuthus sobotniki* (Kovařík, 2003)
15. *Hottentotta jayakari* (Pocock, 1895)
16. *Hottentotta khoozestanus* (Navidpour et al 2008)
17. *Hottentotta lorestanus* (Navidpour et al. 2010)
18. *Hottentotta saulcyi* (Simon, 1880)
19. *Hottentotta schach* (Birula, 1905)
20. *Hottentotta zagrosensis* (Kovařík, 1997)
21. *Iranobuthus krali* (Kovařík, 1997)
22. *Kraepelinia palpator* (Birula, 1903)
23. *Liobuthus kessleri* (Birula, 1898)
24. *Mesobuthus caucasicus* (Nordmann, 1840)
25. *M. eupeus* (C. L. Koch, 1839)
26. *Mesobuthus macmahoni* (Pocock, 1900)
27. *Mesobuthus phillipsi* (Pocock, 1889)
28. *Mesobuthus vesiculatus* (Pocock, 1899)
29. *Odontobuthus bidentatus * (Lourenco et Pezier, 2002)
30. *Odontobuthus doriae* (Thorell, 1876)
31. *Odontobuthus odonturus* (Pocock, 1897)
32. *Orthochirus farzanpayi* (Vachon and Farzanpay, 1987)
33. *Orthochirus fuscipes* (Pocock, 1900)
34. *Orthochirus gruberi* (Kovařík et Fet, 2006)
35. *Orthochirus iranus* (Kovařík, 2004)
36. *Orthochirus scrobiculosus* (Birula, 1900)
37. *Orthochirus stockwelli* (Lourenço and Vachon, 1995)
38. *Orthochirus varius* (Kovařík, 2004)
39. *Orthochirus zagrosensis* (Kovařík, 2004)
40. *Polisius persicus* (Fet et al. 2001)
41. *Razianus zarudnyi* (Birula, 1903)
42. *Sassanidotus gracilis* (Birula, 1900)
43. *Sassanidotus zarudnyi* (Birula, 1903)
44. *Vachoniolus iranus* (Navidpour et al. 2008)
Scorpionidae
45. *Scorpio maurus townsendi* (Pocock, 1900)
Hemiscorpiidae
46. *H. lepturus* (Peters, 1862)
47. *Hemiscorpius acanthocercus* (Monod et Lourenço, 2005)
48. *Hemiscorpius enischnochela* (Monod et Lourenço, 2005)
49. *Hemiscorpius gaillardi* (Vachon, 1974)
50. *Hemiscorpius persicus* (Birula, 1903)
Diplocentridae
51. *Nebo henjamicus* (Francke, 1980)

Although considerable epidemiological studies on scorpion stings have been done in several countries, such as Morocco ( Abourazzak et al. 2009 ), Tunisia ( Bouaziz et al. 2008 ), Algeria ( Laïd et al. 2012 ), Iran ( Dehghani et al. 2010 ), Brazil ( Vasconcelos et al. 2004 ), and Mexico ( Chowell et al. 2005 ), there is little information regarding this phenomenon that relates the seasonal activity of scorpions to changes in numbers of scorpion stings. This is also true in Iran, and despite high numbers of scorpion stings in the south and southwest of Iran, there is little information regarding the relationship between scorpion stings and meteorological data. The information about scorpion envenomation in Iran is focused on epidemiological, clinical, and paraclinical studies, and in spite of the rich biodiversity of scorpions and their medical importance in Khuzestan Province, there is little information of climatological effects on scorpion incidence ( Taj et al. 2012 ).

Time series analysis is a sophisticated statistical method that is used for data that have been observed over time. Data points in time series are correlated, and statistical inferences are performed that include the correlation structures. The main aim of time series analysis is to describe the behavior of data points over time in a statistical model framework. Therefore, the present systemic survey of scorpion sting data was conducted from the point of view of entomo-meteorological relationships and analyzed statistically for the Dezful area in Khuzestan, southwest Iran. This may enable the local authorities to predict the new outbreak of scorpion stings and help make plans to reduce scorpion stings among the residents of the region.

## Materials and Methods

The place of study was Dezful, with coordinates of 32° 22′57″ N, 48° 24′07″ E in Khuzestan Province, southwest Iran. Data were the number of scorpion stings reported in health centers that were registered monthly during 2007–2011. The monthly scorpion sting reported was examined, and data consisted of 60 observations from March 2007 to February 2011 acquired from the health center division located in Dezful County, one of the earliest health centers in Khuzestan Province. The meteorological data, including average monthly temperature, average monthly humidity, wind velocity, and monthly sunlight hours, were provided by the meteorological office of Dezful County.

They are selected based on our experiences and some epidemiological studies in Khuzestan Province and other provinces of Iran ( Taj et al. 2012 , 2013 ; Vazirianzadeh et al. 2013a , 2013b ; Chowell et al. 2005 ). These authors have examined the effects of climatological variables on incidence of scorpion stings in different parts of Iran and Mexico. The factors include the evaporation (millimeter), average of monthly temperatures (°C), the average of monthly relative humidity (percentage), speed of wind, and sunshine hours.

All the authors have confirmed that temperature (°C) of the environment was the most effective factor. The time series analysis was implemented using MINITAB version 16 statistical software packages. The time series consisted of sequential data points taken at equal increments over time. A broad class of time series model is autoregressive and moving average ARMA ( *p* , *q* ), which in turn comprises three models autoregressive (AR), moving average (MA), and ARMA together. An ARMA ( *p* , *q* ) is given as follows: $\left(\text{1}-\sum_{i=1}^{p}\varphi_{i}B^{i}\right){\text{y}}_{\text{t}}=\left(\text{1}-\sum_{i=1}^{q}\theta_{i}B^{i}\right)a_{t}$ , where *B* is the backshift operator ( *B ^i^ y _t_* = *y _t_*_−_ *_i_* ), are the parameters of ARMA models, respectively. *yt* is our measurement, where *y* denotes the number of scorpion stings and *t* denotes the error term, which assumed normal distribution with mean 0 and constant variance 2. *p* and *q* are the order of AR and MA, respectively.

Autocorrelation function and partial autocorrelation function are two important and useful instruments to identify *q* and *p,* respectively, in the order of the time series model. The Box–Jenkins method ( Brockwell and Davis 2002 ) was used to make the model, and it consisted of three steps, the first is the identification of *p* and *q* , the second is the estimation of parameters, and the third is the verification of the model adequacy. The seasonal activity information of scorpion stings has been performed as percentage records in the related tables.

## Results

In total, 3,755 scorpion sting files from Dezful health centers were monitored from April 2007 to September 2011 in the time series analysis in this study. However, 3,272 files from April 2007 to September 2011 are interpreted in this study with specific regard to seasonal scorpion sting. The results of this study indicated that most cases of scorpion stings took place in the summer, with 1,241 cases (37.98%), followed by spring, fall, and winter with 1,028 (31.42%), 788 (24.08%), and 215 (6.57%) cases, respectively.

**Fig. 1.**
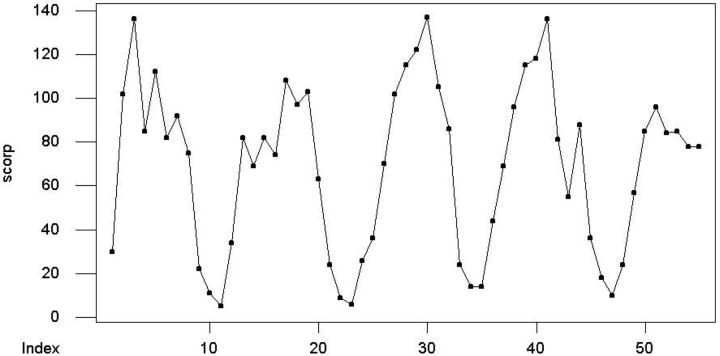
TSP of scorpion stings during 2007–2012.

**Fig. 2.**
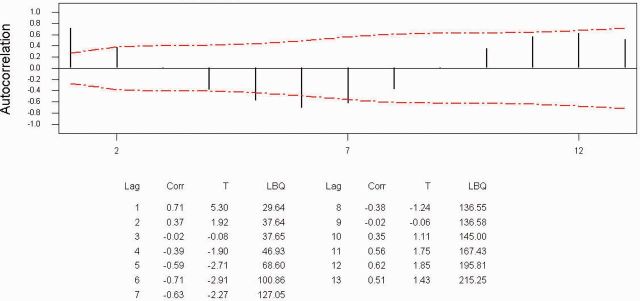
Autocorrelation function for scorpions.

**Fig. 3.**
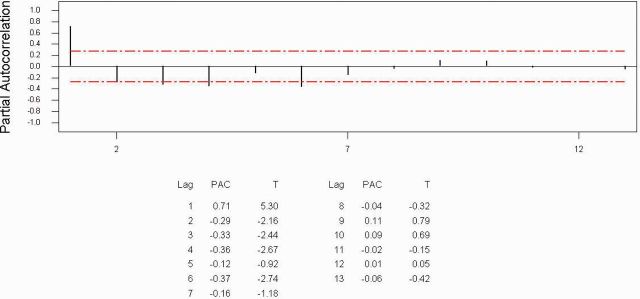
Partial autocorrelation function for scorpions.

Drawing a time series plot (TSP) is the first step in time series analysis. A preliminary concept of the behavior of data is given by TSP. Figure 1 shows the TSP of scorpion sting over time, and there is no increasing or decreasing pattern over time, so the data have stochastic behavior through time. Figures 2 and 3 suggested the order of the model, and both figures suggest *p* = *q* = 2. We carried out ARMA (2, 2) and ARMA (2, 1), and the estimation of parameters is given in Table 2 .

**Table 2.** Final estimates of parameters from ARMA (2, 2) and ARMA (2, 1)

Type	Coefficient	SE coefficient	*T*	*P*
*ARMA (2, 2)*				
AR 1	1.6326	0.0756	21.59	0.000
AR 2	−0.8527	0.0744	−11.45	0.000
MA 1	1.0530	0.0872	12.07	0.000
MA 2	−0.0556	0.0532	−1.04	0.301
Constant	14.9997	0.0488	307.46	0.000
Mean	68.1507	0.2217		
Modified Box–Pierce (Ljung–Box) chi-square statistics				
Lag	12	24	36	48
Chi-square	8.4	24.4	37.3	57.7
df	7	19	31	43
*P*	0.301	0.182	0.201	0.067
*ARMA (2, 1)*				
AR 1	1.6246	0.0749	21.70	0.000
AR 2	−0.8503	0.0749	−11.36	0.000
MA 1	0.9875	0.0438	22.52	0.000
Constant	15.3167	0.0557	275.11	0.000
Mean	67.8586	0.2467		
Modified Box–Pierce (Ljung–Box) chi-square statistics				
Lag	12	24	36	48
Chi-square	9.2	25.1	40.0	61.3
df	8	20	32	44
*P*	0.329	0.197	0.156	0.043

The MINITAB result of ARMA (2, 2) shows that the second order of MA (2) is not significant ( *P* value = 0.301); therefore, we performed ARMA (2, 1) as it is given above and all parameters are significant, so the second one is suggested as the final model. Furthermore, Ljung–Box results are given for both models that suggest no seasonal pattern in models.

**Fig. 4.**
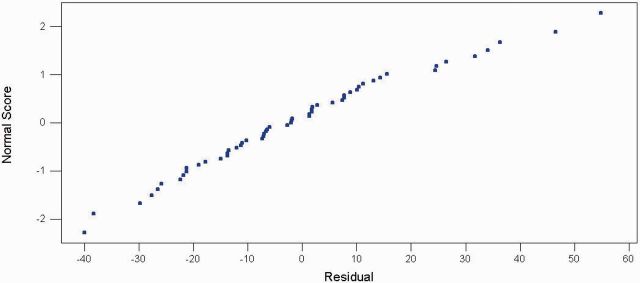
Normal probability plot of the residuals (response is scorpion).

**Fig. 5.**
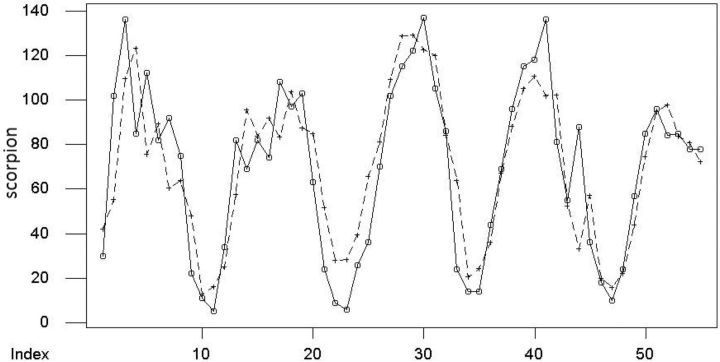
Observations (O) and fitted values (+).

The last step is adequacy evaluation of the model. Figure 4 shows that residuals generated from ARMA (2, 1) follow a normal distribution because the residual points lie approximately on a straight line. The second evidence of model adequacy is shown in Figure 5 . The fitted values and observations are depicted in this figure, and the figure shows that the fitted values are a proper estimator of observations.

Finally, multiple regressions are used to verify the relationship between climatology variables and scorpion stings. Four climatology variables comprising the monthly averages of temperature, humidity, wind velocity, and sunlight hours were considered as explanatory variables and scorpion sting as the response variable. These results showed that temperature had significant effects on scorpion sting. Table 3 presents the coefficients of the multiple regression model indicating that approximately five cases of scorpion stings increased for each unit increase in temperature. Wind velocity and sunny hours had indirect relationships with scorpion sting and were not statistically significant. In addition, the temperature increased parallel to lower relative humidity, greater sunny hours, and higher rate of evaporation.

**Table 3:** 

Predictor	Coef	SE coefficient	*T*	*P*
Constant	−98.56	43.80	−2.25	0.029
Temperature	5.4181	0.9491	5.71	0.000
Humidity	0.7770	0.4389	1.77	0.083
Wind velocity	−0.2563	0.6977	−0.37	0.715
Sunshine hours	−0.04080	0.09512	−0.43	0.670

Scorpion = −98.6 + 5.42, temperature: + 0.777, humidity: −0.256, wind velocity: −0.0408; sunshine hours *S* = 18.46, R-Sq = 78.6%, R-Sq (adj) = 76.9%.

Also ∼8 (7.7%) cases of scorpion sting increased as humidity increased by 10%. The R-sq of the model was 78.6%, which indicates that the selected climatology variables could explain 78.6% of the total observation of scorpion sting through the above multiple regressions.

## Discussion

The data show that cases of scorpion sting have a correlated structure over time; therefore, the time series model is an appropriate model to describe the behavior of data over time as an epidemiological study. ARMA (2, 1) shows that the cases of scorpion sting at each time can be estimated as two last successive cases as AR (2) and the last MA term MA (1). The MA term expresses some stochastic factors occurred at the previous step, which were not determined, but their effects were estimated by the MA part in the time series model. Moreover, the cases of scorpion sting could be forecast through the time series model that creates constructive tools for monitoring and managing of health.

It is well documented that scorpion stings are a multifactorial phenomenon in the world. These factors are geographical locations, socioeconomically structure, species of scorpions, and meteorological data ( Ozkan et al. 2008 ). However, the effects of the last factor have been discussed rarely in scorpion sting incidence, but recently, climate data have been used to monitor and predict the outbreaks of different diseases, including arthropod-borne diseases, by epidemiologists and ecologists ( Chowell et al. 2005 ).

This research is based on the retrospective data. Therefore, the interpretation of relationships between scorpion sting incidence and climatic factor is not possible based on the species. The scorpions that have been brought by the patients to the Dezful health centers were identified as *Androctonus crassicauda* , *H. lepturus,* and *Mesobuthus eupeus* . The scorpion species were identified using Farzanpey’s (1987) keys to the Iranian scorpions. This is in accordance with other parts of Khuzestan. Therefore, to explain the seasonal activity of medical importance, we rely on the other study that was carried out in Ramhormoz of Khuzestan.

*H. lepturus* , *A. crassicauda* , and *M. eupeus* are the main species responsible for stings in Khuzestan ( Chitnis et al. 1993 , Pipelzadeh et al. 2007 ), but *H. lepturus* is the most venomous of all types of scorpions in the region and contributes to 95% of all mortalities in scorpion-stung patients. The results of Mohseni et al.’s (2013) study in Ramhormoz (31° 17′0″ N, 49° 36′0″ E) showed that the maximum activities of *H. lepturus* were in May and August for A *. crassicauda* . This is confirmed by the results of Vazirianzadeh and Samie (2006) , who also reported that the maximum activities of *H. lepturus* were in May and August for *A. crassicauda* . Therefore, the more important scorpionism in the mild temperate seasons is due to *H. lepturus* stings and to *A. crassicauda* during warmer seasons. With respect to the habitats of scorpions and place of scorpion sting, a 5-year-period study in the Dezful area, which is situated in Khuzestan Province, showed that 58% of cases occurred in rural areas and 42% in urban areas. There was no remarkable change during 5-year studies in Dezful from the point of rural or urban scorpion stings, and each year had a constant trend in either a rural or an urban area. This confirms the results of this study, which shows 78.6% of the observed variance was based on climatological factors. It is assumed that scorpionism in the Dezful area is a rural event. However, on the basis of González-Sponga (1996) , the scorpion sting may be increased in peripheral areas of cities due to ecological alterations during the urbanization process of areas naturally occupied by scorpions, especial nonburrowing scorpions in Dezful. It is important that all three recognized scorpions in Dezful health centers are nonburrowing animals. However, the study of Vazirianzadeh et al. (2008) has introduced scorpion sting as an urban event in Ahvaz, the capital of Khuzestan, according to González-Sponga (1996) .

Taj et al. ( 2012 , 2013 ), Chowell et al. (2005) , and the authors of this study have stated that environmental temperature (°C) was the essential ecological factor regarding biology of scorpions, and this fact resulted in increasing scorpion sting incidence with a corresponding increase in the environmental temperature (°C) because scorpions are cold-blooded animals.

However, Taj et al. (2012) have explained that there was a negative significant correlation between scorpion sting incidence and mean of environment relative humidity ( *r*^2^ = −0.855 and *P* value = 0.0001). This occurred in the months of January and February with the least frequent scorpion sting and the highest environment relative humidity during 2006–2008 in Ramshir (30° 53′28″ N, 49° 24′21″ E, south of Khuzestan Province). This fact is confirmed by Taj et al. (2013) in their Baghmalek (31° 302 N, 49° 552 E, east of Khuzestan Province) study, which found a negative significant correlation between scorpion sting incidence and mean of environment relative humidity ( *R*^2^ = −0.917 and *P* value = 0.0001). This happened in August, with the most frequent scorpion sting and the least environment relative humidity during 2007–2009 in Baghmalek. This was confirmed by recording the maximum environment temperature in Baghmalek, which was 33.3°C in August, as the warmest month of the year during 2007–2009. In addition, studies by Taj et al. ( 2012 , 2013 ) have explained that the rates of temperature (°C), sunshine hours, and evaporation (millimeter) confirm each other with respect to the incidence of scorpion stings in Ramshir and Baghmalek counties. In contrast, only the rate of temperature (°C) was a significant factor regarding incidence of scorpion sting in Dezful in this study. However, it is most important that the scorpion stings take place throughout the year with respect to all the aforementioned studies.

To the best of our knowledge, this study is the first attempt to monitor statistically the effects of climatologically variables on incidence of scorpion stings in humans in an endemic region of Iran. However, there are several published articles that have discussed the seasonal or geographical distribution data as an epidemiologic risk factor in the scorpion sting outbreaks in Iran but without statistical analysis (Dehghani et al. 2005, Pipelzadeh et al. 2007 , Sedaghat et al. 2011 ).

Data collected in this study revealed that the highest incidence of scorpion sting cases took place in summer (37.98%) during 2007–2011 in Dezful County. This is consistent with other studies of southwest Iran ( Chitnis et al. 1993 , Vazirianzadeh and Samie 2006 ), Saudi Arabia ( Al-Sadoon and Jarrar 2003 , Jarrar and Al-Rowaily 2008 ), and Turkey ( Ozkan and Kat 2005 , Ozkan et al. 2006 ). These authors have reported that 49.7–93.4% of scorpion sting cases occurred in summer. There are two reasons behind this fact: 1) scorpions are ectothermal animals; therefore, their activities increase with the increase of the environmental temperature. It is assumed that the warmer seasons are the time of reproduction for scorpions ( Cala-Riquelme and Colombo 2010 ). However, in the tropics, scorpions are active all year long, although they are more active during the hotter months ( Spirandeli-Cruz 1994 ). 2) The sociocultural points are regarded after harvest treatments of the crops, such as bundling of vegetables or packaging the other crops of the area, and using outdoor areas of houses as sleeping places during summer. Both reasons increase the contacts of scorpions with humans, which leads to more envenoming in the Dezful area during summer ( Mohseni et al. 2013 ). However, these results are in contrast to the study of Lordegan, southwest Iran ( Vazirianzadeh et al. 2013b ), in which the rate of scorpion stings was highest in spring (49.72%). These differences were presumably due to the variation of geographical, climatological, and species distributions ( Chowell et al. 2005 , Dehghani et al. 2010 ).

A considerable point of this study was using numbers of scorpion stings as an indirect method to predict scorpion population and to determine the scorpion seasonal activities in Dezful County. Indirect methods may be useful in assessing damage in some situations ( Timm 1979 ).

There is a consistency between the obtained results of this study in Dezful and the results of different studies in Ramshir and Baghmalek of Khuzestan and Sirjan of Kerman provinces ( Taj et al. 2012 , Vazirianzadeh et al. 2013a ) regarding average monthly temperature. Taj et al. (2012) have indicated that the incidence of scorpion stings is a climatologically dependent phenomenon in Ramshir. They have observed that the correlation rates between climatology data, including average monthly temperature, relative humidity, and sunlight hours, and incidence of scorpion stings were statistically significant but not significant with speed of wind. This contradicts the reports of local residents, who believe that when the wind blows, the rate of scorpion stings increases.

However, the significance was positive for all except the correlation between incidence and relative humidity ( Vazirianzadeh et al. 2013a ), and in their study, the same results have been obtained as in the study by Taj et al. (2012) . However, the authors of this study have observed this positive significant correlation only regarding incidence of scorpion stings and average monthly temperature in Dezful. We did not find any significant relationship between scorpion sting incidence and other climatology data. This is consistent with the results of Chowell et al. (2005) in Mexico regarding average monthly environment temperature. This relationship explains that activity of scorpions is dependent on the environmental temperature, as would be expected for poikilothermic arthropods ( Yip and Hui 1989 ).

The resulting predictive for the scorpion sting incidence in this region explains 78.6% of the observed variance. This indicates that the scorpion activity in Dezful County is highly associated with temperature changes.

The data obtained during this study showed that the majority of rainfall in Dezful occurred in the cold and temperate months, which corresponded with the least number of scorpion sting cases during the year. In contrast, the majority of scorpion sting cases occurred in the months when there was no rain. Therefore, the theory that indicates there are more scorpion sting cases during rainy periods because of flooding does not work in the Dezful area. Then, our statistical approach that nominated the environmental temperature as the main climatological factor related to the number of scorpion sting cases in the studied area is confirmed, indirectly. This climatologic trend happens in the majority of regions in Khuzestan Province.

There could be sociological, behavioral, or economic factors that were not included in the recent study ( Chowell et al. 2005 ) that may affect the incidence of scorpion stings.

From the data of this study, it is concluded that the scorpion activity in Dezful County is an environmental climatology-dependent phenomenon. Therefore, the temperature is the essential factor, which could be used in the prediction modeling of scorpion sting incidence. The socioeconomic status of the population is another important factor that should be considered in future studies.
